# What a Trainee Surgeon Should Know About Refeeding Syndrome: A Literature Review

**DOI:** 10.7759/cureus.2388

**Published:** 2018-03-28

**Authors:** Muneeba Nasir, Balakh S Zaman, Ahmad Kaleem

**Affiliations:** 1 Mayo Hospital, King Edward Medical University, Lahore, PAK

**Keywords:** refeeding syndrome, trainee surgeon, review

## Abstract

Refeeding syndrome (RFS) is potentially fatal, yet there is limited understanding regarding its management among general surgeons due in part to a lack of universally accepted guidelines for RFS diagnosis. The aim of this review is to equip general surgery trainees with the essentials of RFS including a review of the National Institute for Health and Care Excellence (NICE) best practice guidelines for RFS. We used the keywords “refeeding”, “syndrome”, and “hypophosphatemia” to search PubMed, Embase, and Medline databases. We reviewed approximately 130 indexed papers for relevance. Having profound knowledge of nutritional needs in critically ill patients will help trainee surgeons prevent illnesses in the spectrum of RFS, and, over time, this would immensely contribute to reducing the morbidity and mortality associated with RFS.

## Introduction and background

The concept of refeeding syndrome (RFS) was first established in the Second World War. Prisoners of war previously held in the Far East developed neurological and cardiac abnormalities precipitated by the rapid institution of nourishment [1]. RFS, which is potentially fatal, is defined as “significant fluid and electrolyte abnormalities pertaining to metabolic disturbances following rapid oral, enteral or parenteral refeeding in chronically undernourished patients” [2]. The most profound biochemical abnormality is hypophosphatemia, while other biochemical abnormalities include a disturbance of sodium and fluid balance; a deficiency of thiamine; hypomagnesemia; hypokalemia; and changes in protein, fat, and carbohydrate metabolism [3].

The data relevant to the incidence of RFS are deficient mainly because there is no universal consensus on one single definition of RFS. In a thoroughly designed prospective study conducted on a heterogeneous group of intensive care patients, nearly 34% of them developed hypophosphatemia soon after the commencement of feeding [4]. In 2010, a study conducted by the National Confidential Enquiry Into Patient Outcomes and Death of the United Kingdom on 877 records of adult patients who received total parenteral nutrition showed that 14.7% of those at high-risk of RFS developed stigmata of RFS with hypophosphatemia being the most significant biochemical abnormality [5]. Recently, in 2013, a national audit of parenteral nutrition cases conducted in six hospitals in New Zealand revealed RFS documented in 17.6% of the cases [6]. All these studies illustrate the need for a thorough understanding of RFS by clinicians at all levels.

There is currently limited understanding of RFS and its management among general surgeons due in part to a lack of universally accepted, internationally recognized guidelines for the detection and diagnosis of RFS [7]. As a result, surgeons are often not aware of this problem, and many patients who are at high risk of developing RFS are not managed in specialist care. Another alarming hurdle is the morbidity and mortality associated with RFS are not well documented, with only Level 2 and Level 3 evidence available in the literature. The aim of this review is to equip general surgeon trainees with the essentials of RFS awareness including a review of the National Institute for Health and Care Excellence (NICE) guidelines for best practices for the early recognition of RFS, given that prophylaxis is the best way to reduce morbidity and mortality associated with this underdiagnosed and undertreated condition.

## Review

We used the keywords “refeeding”, “syndrome”, and “hypophosphatemia” to search PubMed, Embase, and Medline databases for relevant articles. We cross-checked our findings with a personal reference list and books. We reviewed approximately 130 indexed papers for relevance. The quality of evidence in the published articles was ascertained in accordance with the 2009 Levels of Evidence from the Oxford Centre for Evidence-Based Medicine [8].

Pathogenesis: How does RFS develop?

**Figure 1:**
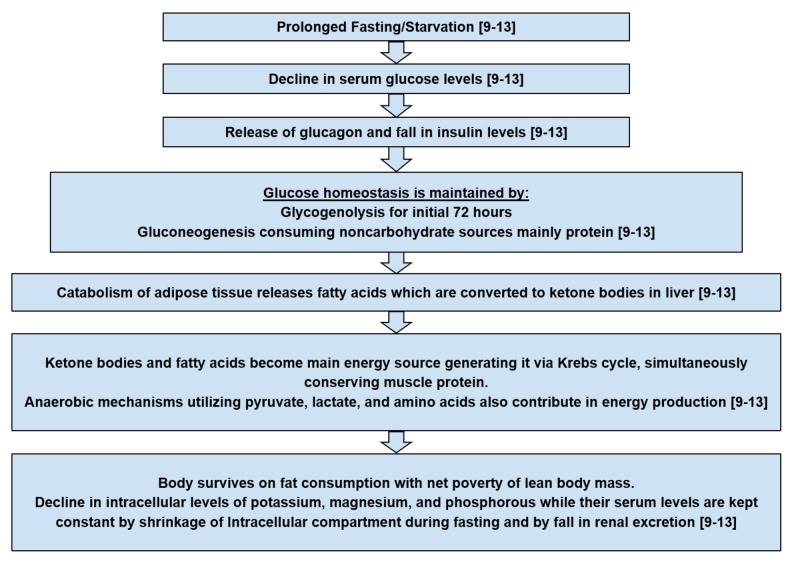
Pathogenesis of refeeding syndrome - flow chart

**Figure 2:**
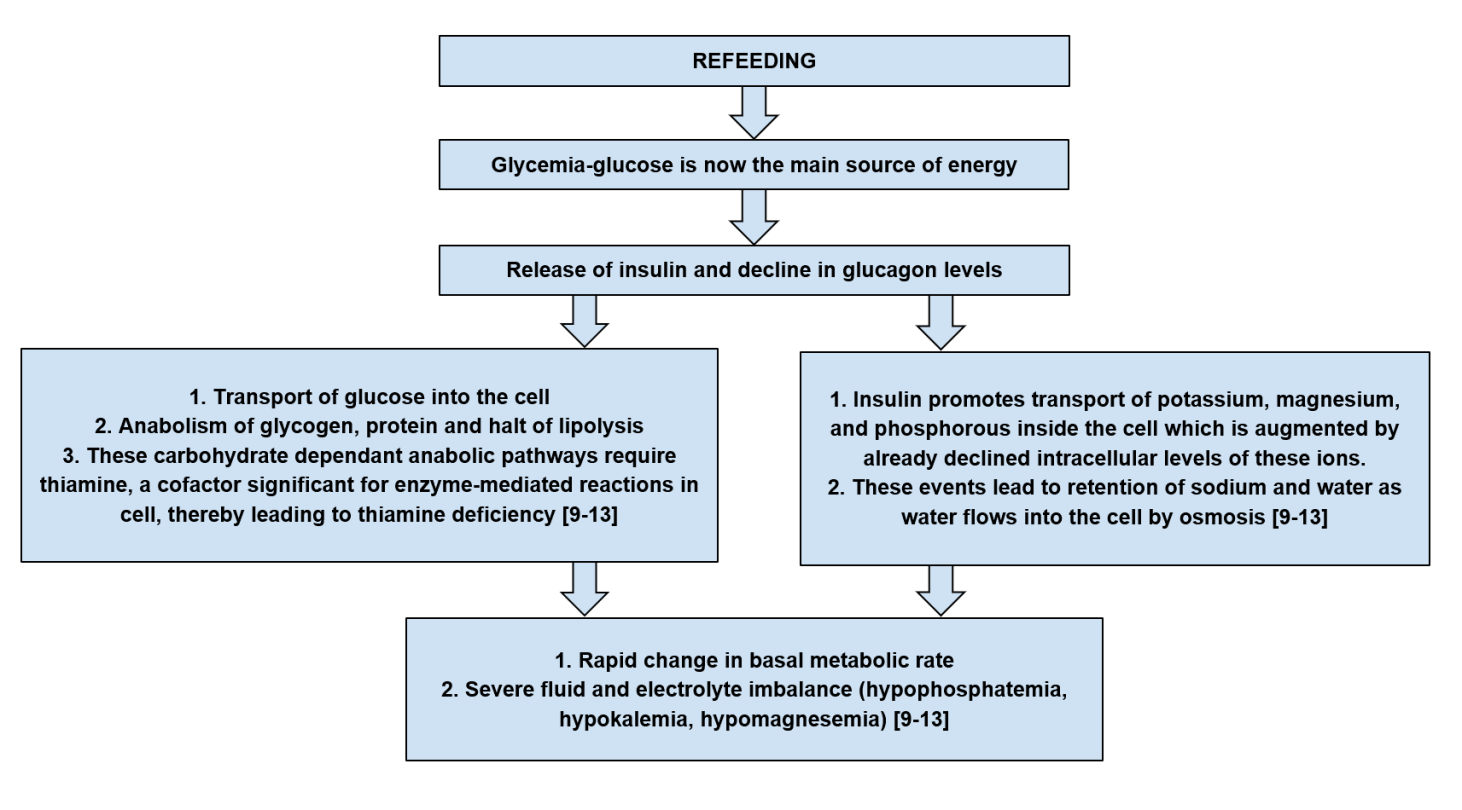
Metabolic and hormonal changes leading to refeeding syndrome

It is essential to understand the pathophysiological events causing metabolic and hormonal disturbances that lead to RFS to have a clear understanding of the tools necessary for its prevention. The flowcharts in Figure 1 and Figure 2 give a thorough overview of RFS pathogenesis [9-13].

Risk factors for developing RFS

One of the pre requisites of developing RFS is chronic malnutrition. According to a study conducted by Hies et al. in 2005, nearly one-third to half of the patients admitted in the hospital are undernourished [14]. Table 1 provides the NICE guidelines for identifying patients at risk of acquiring RFS, which are a surgeon’s best ally [15].

**Table 1:** National Institute for Health and Care Excellence - guidelines for identifying patients at high risk of refeeding syndrome

One Or More of The Following Symptoms	Or	Two Or More of The Following Symptoms
Body mass index < 16 kg/m2		Body mass index < 18.5 kg/m2
Unintentional weight loss > 15% in last six months		Unintentional weight loss > 10% in last three to six months
Little or no nutritional intake for past 10 days		Little or no nutritional intake for > 10 days
Low levels of potassium, phosphate or magnesium prior to feeding		A history of alcohol misuse or drugs including chemotherapy, insulin, diuretics or antacids

Other predominant risk factors of RFS are listed in Table 2, indicating the association between RFS, comorbid conditions, and nutritional status [7,16-17].

**Table 2:** Patients at high risk of developing refeeding syndrome

Patients with anorexia nervosa [7]	Elderly patients (comorbid conditions) [7,16]
Patients with chronic alcoholism [15]	Uncontrolled diabetes mellitus [16]
Oncology patients [16]	Chronic pancreatitis [7]
Chronic malnutrition (marasmus, under profound stress and undernourished > seven days, Malabsorptive disease) [15]	Long-term use of antacids (as magnesium and aluminium bind phosphate) or diuretics (electrolyte imbalance) [15]
Postoperative patients [7]	Acquired immune deficiency syndrome [7]

Clinical manifestations of RFS

The clinical spectra of RFS are reflective of disturbances in serum electrolyte and micronutrient levels affecting cell membrane potential and thereby impairing function in cardiac, nerve, and muscle cells. The severity of symptoms depends upon the extent of biochemical derangements and range from mild complaints of nausea and vomiting to life-threatening respiratory insufficiency and cardiac arrest. The clinical picture of RFS is highly unpredictable [4].

The clinical consequences of biochemical inadequacies in RFS are listed in Table 3 [18-19].

**Table 3:** Clinical consequences of micronutrient abnormalities in refeeding syndrome WBC: white blood cell.

Micronutrient	Etiology of Micronutrient Deficiency	System Affected by Micronutrient Deficiency	Clinical Consequences
PHOSPHATE [18-19]	1. Redistribution of cellular phosphate	Cardiovascular	Arrhythmia, cardiomyopathy, heart failure, hypotension
	2. Loss from renal tubules	Neurological	Convulsions, Altered sensorium, tetany, delirium
	3. Deficient intake	Renal	Metabolic acidosis, Acute Kidney Injury
	4. Septic State	Hematological	Hemolysis, decreased platelet count, WBC dysfunction
	5. Liver pathology	Skeletal	Myalgia, rhabdomyolysis, weakness of the diaphragm
		Endocrine	Insulin resistance, hyperglycemia, osteomalacia
THIAMINE [18-19]	1. Increased cellular usage	Neurological	Wernicke-Korsakoff syndrome (confusion, ataxia, psychosis)
		Cardiovascular	Beriberi disease, congestive cardiac failure
		Skeletal	Myalgias
POTASSIUM [18-19]	1. Redistribution of ions	Cardiovascular	Ventricular arrhythmias, hypotension, cardiac arrest,
	2. Loss of potassium from kidneys		Tachycardia/bradycardia
	3. Loss of potassium from gut	Respiratory	Hypoventilation, respiratory center depression
		Metabolic	Metabolic alkalosis
		Skeletal	Muscle fatigability and twitching
		Gastrointestinal	Nausea, emesis, diarrhea, paralytic ileus
SODIUM [18-19]	1. Changes in serum osmolality in the central nervous system	Cardiovascular	Arrhythmias, heart failure
		Renal	Renal failure
		Respiratory	Pulmonary edema, hypoventilation
		Skeletal	Edema, muscle cramps
MAGNESIUM	1. Redistribution of cellular ions	Cardiovascular	Arrhythmias (paroxysmal ventricular or atrial)
	2. Deficient Intake	Respiratory	Decreased ventilation, respiratory failure
	3. Enhanced Renal losses	Gastrointestinal	Anorexia, diarrhea, vomiting, constipation
	4. Diabetes mellitus	Neuromuscular	Myalgia, muscle cramps, seizures, depression, hallucinations
	5. Hyperaldosteronism	Others	Anemia
	6. Alcoholism		
	7. Hyperthyroidism		

How could clinicians prevent RFS in hospitalized patients?

The fundamental tool to successfully manage RFS is to prevent it by preferably adopting a multidisciplinary approach [20]. There are five basic approaches to prevent the development of RFS in hospitalized patients consisting of the early recognition of patients at high-risk of acquiring RFS; meticulous clinical and biochemical monitoring following the institution of refeeding in individuals in high-risk groups [18]; designing appropriate feeding regimens as seen in Table 4; screening patients at risk of developing RFS at the time of hospital admission; and working with dieticians and nutritional team members to devise individualized refeeding strategies for each patient [21].

Refeeding protocols in patients at high risk of developing RFS

According to NICE, clinicians should commence refeeding at no greater than 50% of the energy needs in patients who have very little or no intake in the previous five days, and feeding rates should be tailored according to the patient’s clinical response [15]. In all high-risk groups, nutrients need to be repleted slowly at 0.042 MJ/kg/day and are increased to fulfill or exceed needs in the following four to seven days [15]. In severely malnourished patients (i.e., those with a body mass index less than 14 kg/m2 or a minimal intake for more than 14 days), it is advised to institute refeeding at 0.021 MJ/kg/day with rigorous cardiac monitoring [15] Concurrent prophylactic supplementation of vitamins, restoration of fluid balance, and correction of biochemical disturbances are also recommended. Table 4 demonstrates refeeding regimens [7,15,18,22].

**Table 4:** Refeeding protocols in patients at high risk of developing refeeding syndrome *Strength of the recommendation (i.e., a and b) cannot be commented upon due to lack of randomised control trials conducted on RFS. BMI: body mass index; IV: intravenous; NICE: National Institute for Health and Care Excellence.

Macronutrient	Day of Refeeding	Recommendation[a=NICE,b=Stanga et al.]^*^	Prophylactic Micronutrient Supplementation in Tandem with the Institution of Feeding
ENERGY	1st Day	(a) [NICE] Start with 10 kcal/day [15]	(a) Aim to keep "zero balance" by restricting fluids [18]
		(b) [Stanga et al.]If BMI is < 14kg/m2 or starved for > 15 days, initial intake = 5 kcal/kg/day [7]	(b) Intravenous vitamin B complex and thiamine before starting feeding (IV 300 mg when feeding instituted) [22]
			(c) Sodium: 1 mmol/kg/day (restricted) [22]
			(d) Phosphate up to 8 mmol/kg/day [22]
			(e) Potassium: 1-3 mmol/kg/day [22]
			(f) Magnesium: 0.3-0.4 mmol/kg/day [22]
	2nd, 3rd, 4th Day	(a) NICE Increase at 5 kcal/day if minimal tolerance, stop/keep low feeding regimen [15]	(a) Identification and correction of biochemical abnormality if any (see Table 5)
		(b) Stanga et al. 10 kcal/day and by the 4th day, give 15 kcal/day [7]	(b) Continue intravenous thiamine until the 3rd day (100 mg/day as maintenance) [22]
			(c) Rigorous clinical and biochemical monitoring [18,22]
	5th, 6th, 7th Day	(a) NICE Increase at 5 kcal/day) if minimal tolerance, stop/keep low feeding regimen [15]	(a) Monitor renal and liver function tests
		(b) Stanga et al. 20-35 kcal/day [7]	(b) Maintain "zero" fluid balance [22]
			(c) Iron supplementation to be considered by day 7 [22]
			(d) Continue clinical and biochemical monitoring [22]
	8th,9th,10th Day	(b) [Stanga et al.] 30-35 kcal/day or feed to full need [7]	
CARBOHYDRATES	Day 1 to 10	(a) NICE 50% to 60% of total energy requirement [15]	
FATS	Day 1 to 10	(a) NICE 30% to 40% of total energy requirement [15]	
PROTEIN	Day 1 to 10	(a) NICE 15% to 20% of total energy requirement [15]	

Monitoring in tandem with the institution of feeding in patients at high risk of developing RFS

A clinician needs to keep a strict eye on clinical and biochemical parameters as soon as refeeding is started in hospitalized patients at significant risk of acquiring RFS. The meticulous clinical monitoring encompasses blood pressure and heart rate monitoring, strict control of refeeding rate, strict fluid intake/output recording, monitoring fluctuations in patient’s weight, keeping an eye on neurological manifestations, and educating the patient. Biochemical monitoring consists of strictly recording daily serum electrolytes, arterial blood gas, and blood glucose levels, as well as monitoring electrocardiograms in critical cases and maintaining awareness of other energy resources [18,23].

Management of electrolyte and micronutrient inadequacies in RFS

Given the potentially life-threatening outcomes of electrolyte abnormalities in RFS, it is essential to have a universally accepted regimen to correct such imbalances and identify inadequacies early, particularly in thiamine, phosphate, magnesium, potassium, and sodium levels to successfully manage RFS [17]. There is a lack of consensus on the best regimen to replete micronutrient and electrolyte deficiencies in RFS [18]. Following an extensive literature review, we have devised the protocols in Table 5 for the management of electrolyte imbalances in RFS [22,24-25].

**Table 5:** Management of electrolyte and micronutrient disturbances in refeeding syndrome

Electrolyte Deficiency	Correction Protocols
HYPOPHOSPHATEMIA [22,25]	
(a) Mild (0.6-0.8 mmol/L)	1. 0.3-0.6 mmol/kg/day via oral route
	Enhance intake via diet and add multivitamin supplement containing phosphate
(b) Moderate (0.3-0.6 mmol/L)	1. 0.6 mmol/kg infused slowly intravenously over 6 hours or
	2. 2-3.5 g/day via oral route in divided doses
(c) Severe (<3 mmol/L)	1. Slow intravenous infusion at the rate of 1 mmol/kg over 12 hours
HYPOMAGNESEMIA [22,25]	
(a) Mild (0.7 mmol/L)	1. Oral supplementation with magnesium lactate or enhance dietary intake
(b) Moderate (0.5-0.7 mmol/L)	1. Per oral intake of magnesium oxide/citrate (15 mmol/day) or
	2. Intravenous slow infusion of 8-32 mmol/kg (max, 0.5 mmol/kg) with daily infusion of 8 mmol over 2 hours
(c) Severe (0.5 mmol/L)	1. Slow intravenous infusion of 32-64 mmol/kg (max, 1 mmol/kg) with 8 mmol over 2 hours
HYPOKALEMIA [24]	
(a) Mild (3-3.5 mmol/L)	1. Enhance dietary intake (40 mmol/day in 3 divided doses)
(b) Moderate (2.5-3 mmol/L)	1. Oral: 20 mmol in 3 divided doses every 2 hours or
	2. Intravenous: Slow infusion of 10 mmol over 1 hour. Recheck and infuse again if required
(c) Severe (<3 mmol/L)	1. Slow intravenous infusion of (40 mmol) over 2 hours
HYPONATREMIA [24]	
(a) Mild (130-135 mmol/L)	1. Restrict free fluid
(b) Moderate (125-130 mmol/L)	1. Restrict free fluid
	2. Intravenous infusion of half normal saline or saline with correction being done at (1-2 mmol/hour)
(c) Severe (<125 mmol/L)	1. Replace at the rate of 2 mmol/hour with 3% sodium chloride
THIAMINE [22]	1. Intravenous: 300 mg at the institution of feeding
	2. Maintenance dose: 100 mg

## Conclusions

All trainee surgeons taking care of vulnerable patients who might need nutritional supplementation should be able to ascertain the risk of RFS. So far, there has been no randomized controlled trials conducted on this potentially life-threatening syndrome and this is mainly attributable to the ethical restrictions. Instead, treatment is based on observational data. These facts emphasize the significance of having sound knowledge of the careful re-institution of feeding in patients at high risk of developing RFS. Robust actions are needed to determine the incidence and arrive at a consensus on a set of guidelines for the management of RFS. Having profound knowledge of nutritional needs in critically ill, high-risk patients will help trainee surgeons prevent the onset of the illness spectrum of RFS. Prevention of RFS spectrum conditions would significantly contribute to reducing the morbidity and mortality of RFS. Following the review, we define RFS as "clinical spectra of fluid disturbances with either one or all of the biochemical abnormalities (i.e., hypophosphatemia, hypokalemia, hypomagnesemia and/or thiamine deficiency) resulting from metabolic changes precipitated by the rapid institution of enteral/parenteral feeding in chronically undernourished patients". However, robust studies should be conducted to formulate a universal definition of RFS, accurately ascertain the incidence of RFS, and devise optimal refeeding regimens via high-quality, randomized controlled clinical trials.
